# Branched-Chain Amino Acids Metabolism and Their Roles in Retinopathy: From Relevance to Mechanism

**DOI:** 10.3390/nu15092161

**Published:** 2023-04-30

**Authors:** Xiaonan Zhang, Mengxue Xia, Yingjie Wu, Fang Zhang

**Affiliations:** 1 National Clinical Research Center for Eye Diseases, Shanghai General Hospital, Shanghai Jiao Tong University School of Medicine, Shanghai 200080, China; 2 Liaoning Provence Key Laboratory of Genome Engineered Animal Models, National Center of Genetically Engineered Animal Models for International Research, Institute for Genome Engineered Animal Models of Human Diseases, Dalian Medical University, Dalian 116000, China; 3 Shanghai Key Laboratory of Ocular Fundus Diseases, Shanghai 200080, China; 4 Shandong Provincial Hospital, School of Laboratory Animal & Shandong Laboratory Animal Center, Science and Technology Innovation Center, Shandong First Medical University & Shandong Academy of Medical Sciences, Jinan 250021, China; 5 Department of Molecular Pathobiology, New York University College of Dentistry, New York, NY 10010, USA

**Keywords:** retinopathy, branched-chain amino acids, metabolomics, neuroprotection, oxidative stress, inflammatory, glutamate toxicity

## Abstract

Retinopathy is one of the leading causes of irreversible blindness and vision loss worldwide. Imbalanced nutrients play important roles in the pathogenesis and pathophysiology of retinal diseases. Branched-Chain Amino Acids (BCAAs), as essential amino acids, perform a variety of biological functions, including protein synthesis, glucose metabolism, lipid metabolism, inflammation, and oxidative stress in metabolic tissues of diabetes and aging-related diseases. Recently, it has been shown that BCAAs are highly related to neuroprotection, oxidative stress, inflammatory and glutamate toxicity in the retina of retinopathy. Therefore, this review summarizes the alterations of BCAA levels in retinopathy, especially diabetic retinopathy and aging-related macular disease, and the genetics, functions, and mechanisms of BCAAs in the retina as well as other metabolic tissues for reference. All of these efforts aim to provide fundamental knowledge of BCAAs for further discoveries and research on retina health based on the sensing and signaling of essential amino acids.

## 1. Introduction

The latest Global Burden of Disease study reported that there were 43.3 million blind people in the world in 2020: 295 million people suffered moderate and severe vision impairment and 258 million people had mild vision impairment. By 2050, there are expected to be approximately 90% more people with vision impairment and 50% more blind people [1]. Retinopathy, as one of the leading diseases causing visual impairment and blindness, includes diabetic retinopathy (DR), age-related macular degeneration (AMD), and many other retinal diseases [2,3]. It has become a national and even an international issue with unmet strategies and treatments.

DR is the leading cause of blindness in working-age adults [4]. It is clinically divided into non-proliferative DR (NPDR) and proliferative DR (PDR), which is based on the emergence of neovascularization. According to reports, vascular endothelial growth factor (VEGF) promotes neovascularization and regulates the pathophysiology of PDR [5,6]. However, using anti-VEGF drugs as a mainstream treatment method has many shortcomings [7]. More importantly, there is still a lack of treatment for NPDR. In terms of AMD, it is an aging change in the structure of the macular region, which has been the primary cause of vision decline and blindness in the elderly [8,9]. There are generally two types of AMD: dry-AMD and wet-AMD. The former is characterized by focal yellow extracellular deposits, whereas the latter is characterized by neovascularization [10]. Although dry-AMD accounts for almost 80–85% of all AMD patients, there is no effective treatment at present [11]. Current retinopathy treatments are limited for neovascularization, mainly including laser photocoagulation, vitrectomy, and anti-VEGF therapy [12]. New treatment modalities and therapeutic targets are urgently needed.

Nutrients from food are of great significance in the pathogenesis and pathophysiology of retinopathy. Amino acids are reported to be related to retinal diseases [13]. As specific types of amino acids, branched-chain amino acids (BCAAs) are composed of leucine, isoleucine, and valine. BCAAs account for about 35% of the essential amino acids in mammals, although they cannot be synthesized [14]. BCAAs are also relatively ample in the food supplement, comprising approximately 20% of protein intake. BCAAs play important roles in protein synthesis, energy production, inflammation, oxidative stress, and glucose metabolism in the metabolic tissues of diabetes and aging-related diseases [15,16,17]. Currently, BCAA metabolism and retinopathy have been linked in numerous studies [18,19,20]. In this review, we intend to summarize the metabolism of BCAAs, the changes in BCAA levels in retinopathy, especially DR and AMD, and the genetics, functions, and mechanisms of BCAAs in the retina.

## 2. BCAA Metabolism Process

**Figure 1:**
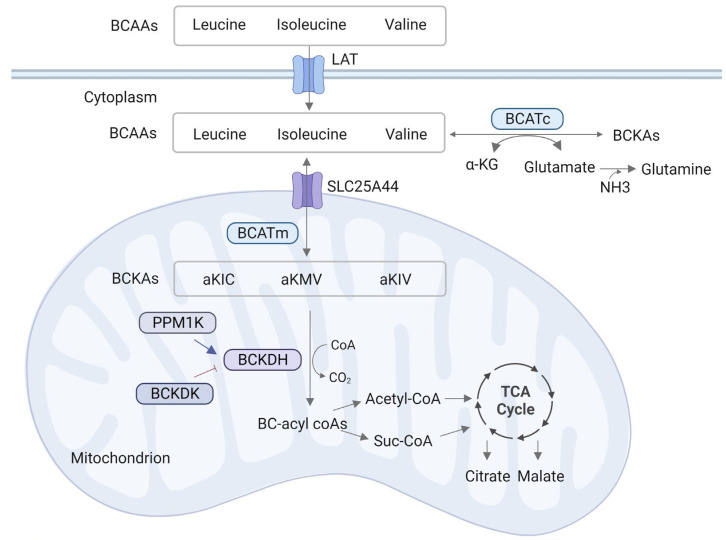
Regulatory factors involved in BCAA catabolism. BCAAs are transported to the cytoplasm by the transporter LAT, then transaminated by BCATc to form BCKA or further transported into mitochondrion by SLC25A44. In the mitochondrion, BCKDK inhibits the activity of BCKDH by phosphorylation, while PPM1K promotes BCKDH activity by dephosphorylation. Eventually, the carbon in BCAAs ends up as CO_2_ or metabolites in the tricarboxylic acid cycle (TCA cycle).

The metabolism process of BCAAs is similar for all life forms, as shown in Figure 1. In mammals, BCAAs initially form branched α-ketoic acids (BCKAs) under the action of transamination by branched-chain amino transferases (BCATs) [21]. To be specific, leucine is converted to α-ketoisocaproic acid (KIC), isoleucine to α-ketomethylvaleric acid (KMV), and valine to α-ketoisovaleric acid (KIV). The most common nitrogen receptor is α-ketoglutaric acid (αKG), which produces glutamate [22]. The reaction is fast and has low variation in free energy, so it may be in equilibrium in most cases. BCAT is encoded by two genes: BCAT1 (or BCATc) encodes cytoplasmic proteins that are expressed primarily in the brain, while BCAT2 (or BCATm) encodes mitochondrial proteins and is widely expressed in various tissues [22,23]. Another enzyme that plays a vital role in BCAA metabolism is the branched amino acid dehydrogenase (BCKDH) complex. As a rate-limiting enzyme of BCAA catabolism, BCKDH is also a key factor in maintaining BCAA homeostasis, and it is present on the mitochondrial inner membrane. Under the catalysis of the BCKDH complex, BCKAs are irreversibly oxidized and decarboxylated, in which CO_2_ is released and coenzyme A (CoA) is covalently added to the oxidized BCKA products [24]. BCKDH is decarboxylated to form the corresponding branched acyl CoA derivatives. Eventually, part of the BCAA carbon is lost in the form of CO_2_, and the other part enters the tricarboxylic acid cycle (TCA cycle) [25]. In addition, BCKDH kinase (BCKDK) or phosphatase (PPM1K), as a key regulatory factor, participates in BCAA metabolism by inhibiting or promoting the activity of BCKDH.

## 3. The Alterations of BCAAs in Retinopathy

Metabolomics is capable of simultaneously identifying and quantifying metabolites in tissues and biofluids [26,27]. The metabolomic analysis elucidates crucial scientific issues related to cell metabolism through the description and characterization of different substrate molecules and then recognizes the pathological processes of diseases via the networks of metabolic profiles [28]. There have been numerous studies applying metabolomics to retinopathy in recent years. Relevant articles were retrieved through searching with the following keywords: (“Branched-chain amino acids” or “Leucine” or “Isoleucine” or “Valine” or “metabolomics”) AND “retina” or “retinopathy” in PubMed before 24 February 2023. Finally, we analyzed nineteen original articles and summarized the species, samples, subjects, platforms, criteria and BCAA levels in these articles (Table 1).

As shown in Table 1, there are mainly three metabolomics analysis platform methods being used to analyze the alteration of BCAAs in retinopathy, namely, nuclear magnetic resonance (NMR) spectroscopy, mass spectrometry (MS), and high-performance liquid chromatography (HPLC) [29,30,31]. To solve the problem of relatively low identification of different metabolites in complex samples, NMR is used in conjunction with different techniques, such as liquid chromatography-MS (LC-MS) and gas chromatography-MS (GC-MS) [32]. Additionally, Frayser et al. [29] and Valle et al. [30] measured BCAA levels using the Beckman Model 121 amino acid analyzer and the Technicon amino acid analyzer, respectively. Masser et al. [31] used a spectrophotometric method to detect BCAA levels.

Various biological samples of retinopathy have been widely detected via metabolomics. In the analysis of studied species, seven of them were from human samples, including plasma and intraocular fluids (subretinal fluid and vitreous); twelve of them were from rodent models, including six from the plasma, retina, and retinal pigment epithelium cells (RPE)/choroid of mice and six from the serum, plasma, retina, vitreous and RPE/choroid of rats, as shown in Table 1. Samples in the remaining study were vitreous humor from pigs. The different samples used for metabolomics analysis had their own characteristics and advantages. Blood was readily available and sampling was minimally invasive to the patient among all clinical samples, therefore serum and plasma were widely used in metabolomics [33]. Serum and plasma can be divided because they are different when it comes to the presence of clotting factors [34]. Intraocular metabolic variations are directly reflected by eye fluids such as vitreous humor and aqueous humor. Located in the posterior section of the eyeball and being the largest structure of the eye, the vitreous is between the retina and the lens. The vitreous is considered a critical structure in ocular diseases such as macular hole formation and the pathogenesis of retinal detachment [35].

BCAA levels were changed in the retinopathy patient’s subretinal fluid (SRF), vitreous, and plasma, as shown in Table 1. Yalcinbayir et al. [36] collected the SRF from patients with rhegmatogenous retinal detachment (RRD) and detected variations in amino acid levels in the SRF. Results showed that the concentrations of BCAAs were decreased. Similarly, the concentrations of amino acids were detected in the subretinal and vitreous fluid of primary RRD patients. Compared to control vitreous, BCAA levels were significantly lower in the SRF of RRD patients [37]. Vitreous samples were collected from twenty-eight patients of type 2 diabetes (T2D) with PDR and twenty-two non-diabetic patients with macular hole. GC-TOFMS was used to discern potential metabolite markers for DR. Results showed that fifteen different metabolites were found in the vitreous, for example, increased levels of BCAAs [38]. Xuan et al. [39] established reliable biomarker models by exploring the abnormal metabolism pathway function of DR as compared with NDR. Their results showed that the levels of leucine, isoleucine, and valine in BCAA metabolism were enhanced in the serum of DR patients. In an analysis of the relationship between metabolite levels of plasma and dark adaptation in AMD, Mendez et al. [40] revealed that BCAA metabolism was associated with rod-intercept time, one measure of dark adaptation. Moreover, Valle et al. [30] found that the levels of BCAAs were diminished in the plasma of four patients with gyrate atrophy (GA) of the choroid and retina. Metabolomics of the plasma from nineteen patients with DR and fourteen controls indicated that BCAA levels were enhanced in the plasma of DR patients, but not significantly [41].

BCAAs were significantly increased in the retinas of DR mice [18,19], DR rats [20,29,42], oxygen-induced retinopathy (OIR) mice [43], and AMD mice [44]. Moreover, leucine and isoleucine were increased in the retina and decreased in the vitreous and RPE/choroid of CNV rats, an AMD model [45]. Xu et al. [46] found leucine and isoleucine were highly enriched in the retina and RPE/choroid, while valine represented a much lower percentage. Consuming more leucine than RPE, the retina showed a low leucine catabolizing level. Leucine also increased the abundance of multiple other amino acids in the RPE/choroid. Except for the retina, BCAAs were significantly increased in the serum [31,47] and plasma [29] of DR rats. Elmi et al. [48] collected vitreous humor from pigs treated with iodoacetic acid (IAA), the porcine model of photoreceptor degeneration. Their results showed that BCAAs were decreased in the vitreous humor in the IAA group, compared to physiological conditions. It was found that BCAA levels were significantly enhanced in human retinal Müller cells cultured after treatment with high glucose.

The above studies found that BCAA levels were altered in variant retinopathy conditions. BCAAs were generally increased in the DR (patients, mice, and rats), AMD (mice, and rats), OIR (mice), and RRD (patients) models, as well as human retina Müller cells with high glucose. BCAAs were reduced in the patients with GA and the pig with photoreceptor degeneration. The reason for this difference may be related to the pathogenesis and process of the disease and still needs to be explored.

**Table 1:** Summary of BCAAs in retinopathy based on metabolomics studies.

Species	Samples	Subjects	Platforms	Criteria	Differential Metabolites	References
Human	SRF and vitreous	46 RRD/7 controls	HPLC	*p* < 0.05	↓ Leucine, Isoleucine, Valine	Yalcinbayir et al. (2014) [36]
	SRF and vitreous	20 SRF and 5 vitreous/10 Vitreous controls	HPLC	*p* < 0.01	↓ Leucine, Isoleucine, Valine	Bertram et al. (2008) [37]
	Vitreous	28 PDR/22 controls	GC-TOFMS	*p* < 0.01	↑ L-Leucine, L-alloisoleucine, L-valine	Wang et al. (2020) [38]
	Plasma	105 NDR, 103 NPDR, 103 MNPDR, 113 SNPDR, and 20 PDR	GC-MS, LC-MS	*p* < 0.05	↑ Leucine, Isoleucine, Valine	Xuan et al. (2020) [39]
	Plasma	19 DR/14 controls	NMR	*p* < 0.05	↑ Leucine	Lin et al. (2019) [41]
	Plasma	4 GA/22 controls	Technicon amino acid analyzer	*p* < 0.05	↓ Leucine, Isoleucine, Valine	Valle et al. (1980) [30]
	Plasma	53 AMD/18 controls	UHPLC-MS	*p* < 0.01	↑ Leucine, Isoleucine	Mendez et al. (2021) [40]
Mice	Retina	10 OIR/10 controls	HPLC-MS/MS	*p* < 0.01	P17: ↑ Leucine, Isoleucine, L-Valine	Zhou et al. (2021) [43]
	Retina	6 DR mice/9 controls	UHPLC-MS/MS, GC-MS	*p* < 0.05	↑ Leucine, Isoleucine	Wang et al. (2022) [18]
	Plasma	DR mice/controls	UHPLC-MS/MS	*p* < 0.05	↑ Leucine, Isoleucine, L-Valine	Gong et al. (2022) [19]
	Retina					
	Retina	6 AMD mice/6 controls	LC-MS	VIP > 1	↑ Leucine, Isoleucine	Natoli et al. (2018) [44]
	Plasma				↑ Leucine, Isoleucine, Valine	
	Retina		GC-MS	*p* < 0.05	Leucine, Isoleucine, Valine	Xu et al. (2020) [46]
	RPE/choroid					
Rats	Retina	DR rats/controls	HPLC	*p* < 0.05	↑ Leucine, Isoleucine, Valine	Gowda et al. (2011) [42]
	Retina	DR rats/controls	HPLC	*p* < 0.01	↑ Leucine, Isoleucine, Valine	Ola MS et al. (2019) [20]
	Retina	6 DR rats/4 controls	Beckman Model 121 amino acid analyzer	*p* < 0.01	↑ Leucine, Isoleucine, Valine	Frayser et al. (1978) [29]
	Plasma					
	Retina	7 AMD rat/6 controls	UHPLC-MS/MS	*p* < 0.05	↑ Leucylleucine, Isoleucylisoleucine	Wei et al. (2022) [45]
	Vitreous				↓ L-Leucine, Isoleucine	
	RPE/choroid				↓ L-Leucine, Leucylleucine, Isoleucylisoleucine	
	Serum	DR rats/controls	Spectrophotometric	*p* < 0.05	↑ Leucine, Isoleucine, Valine	Masser et al. (2014) [31]
	Serum	DR rats/controls	LC	*p* < 0.05	↑ Leucine, Isoleucine, Valine	Gibson et al. (1988) [47]
Pig	VH	13 treated with IAA/17 controls	1H NMR	*p* < 0.01	↓ Leucine, Isoleucine, Valine	Elmi et al. (2019) [48]
Human cell	Human retinal Müller cell	Müller cell with HG or NG	UHPLC-MS/MS	*p* < 0.05	↑ Leucine, Isoleucine, Valine	Gong et al. (2022) [19]

SRF, subretinal fibrosis; RPE, retinal pigment epithelium cells; RRD, rhegmatogenous retinal detachment; DR, diabetic retinopathy; PDR, proliferative DR; NPDR, non-proliferative DR; MNPDR, moderate non-proliferative DR; SNPDR, severe non-proliferative DR; GA, gyrate atrophy of the choroid and retina; AMD, age-related macular degeneration; OIR, oxygen-induced retinopathy; IAA, iodoacetic acid; HPLC, high-performance liquid chromatographic; GC-TOFMS, gas chromatography coupled with time-of-flight mass spectrometry; NMR, nuclear magnetic resonance; UHPLC-MS, ultra-high-performance liquid chromatography–mass spectrometry; HPLC-MS, high-performance liquid chromatography–mass spectrometry; GC-MS, gas chromatography–mass spectrometry; LC-MS, liquid chromatography–mass spectrometry; LC, liquid chromatography; 1H-NMR, 1H Nuclear Magnetic Resonance; p17, mice harvested at seventeen days after birth. ↑, increased; ↓, decreased.

## 4. Biological Functions of BCAAs in the Retina and Other Major Tissues

**Figure 2:**
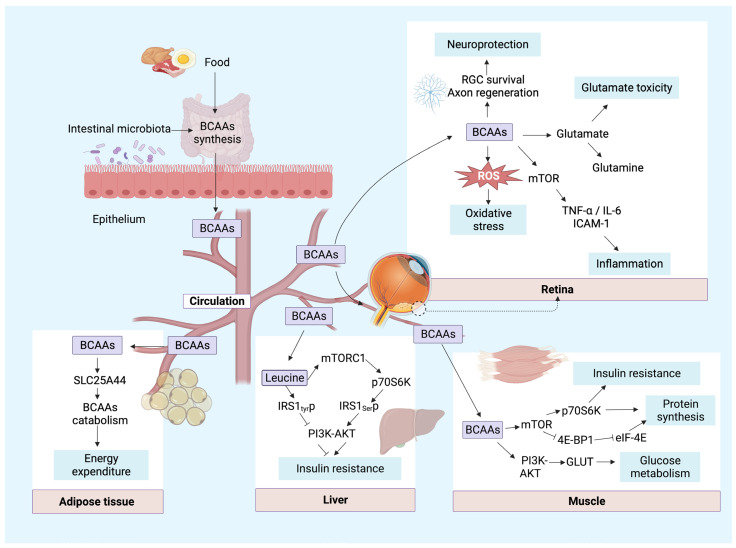
The roles of BCAAs in the retina and other major metabolic tissues. BCAAs from protein diets are absorbed by the intestine and exert crucial functions in the retina and tissues, such as liver, muscle, and adipose tissue. The functions of BCAAs in the retina include regulation of glutamate excitatory toxicity, neuroprotection, oxidative stress, and inflammation. In addition, BCAAs, as critical nutrient substances and signaling molecules, are also involved in the regulation of protein synthesis, glucose metabolism, insulin resistance, and energy expenditure.

BCAAs perform multiple functions in metabolic tissues, including adipose tissue, the liver, and muscle as well as the retina. BCAAs in the retina are mainly absorbed from the small intestine and then transaminated in skeletal muscle, which is the main organ with high activity of BCAT for BCAA metabolism. Oxidative decarboxylation of BCAAs occurs in the liver with the highest BCKDH activity, the heart with higher BCAT activity, and adipose tissue [15,25,49]. Hence, we summarized the biological functions and mechanism of BCAAs in the retina and crucial tissues, such as muscle, liver, and adipose, which may be more conducive to further exploring other functions of BCAAs in the retina (Figure 2).

### 4.1. Regulation of the Excitatory Toxicity of Glutamate

Excitatory toxicity mainly refers to the toxic effects of excitatory neurotransmitter glutamate. Long-term activation of glutamate receptors will lead to a series of neurotoxicity, eventually leading to cell death and the loss of neuron function. As the most abundant amino acid in the central nervous system, glutamate acts as a major excitatory neurotransmitter [50]. Excessive glutamate overstimulates glutaminergic receptors, such as n-methyl-D-aspartic acid and α-amino-3-hydroxy-5-methyl-4-isooxazopropionic acid receptors, and leads to excitatory toxicity. As a result, glutamate removal mechanisms are vital in maintaining the neuron survival of the central nervous system.

BCAAs participate in de novo synthesis of glutamate and determine the maintenance of glutamate levels in the brain, in addition to the glutamine/glutamate cycle [51]. Elevated levels of BCAAs measured in diabetic retinas may lead to increasing glutamate neurotoxic levels through transamination of citric acid (TCA) cycle intermediates. Ola et al. [20] found that excessive BCAAs in retinal Müller cells of diabetic rats led to increased glutamate levels and induced glutamate excitatory toxicity. This result may also be due to increased levels of circulating BCAAs. Contrusciere et al. [52] found that valine and the mixture of BCAAs significantly increased glutamate excitatory toxicity in cortical neurons and was toxic to neurons in the absence of any additional stimulus. This result also suggests that valine, as a major BCAA in glutamate synthesis, may contribute to excitatory toxicity by increasing glutamate availability. In addition, diabetes causes alterations in glutamate metabolism in the retina and may participate in neurodegeneration, especially through glutamate excitotoxicity.

### 4.2. Neuroprotection

In addition to excessive BCAAs causing glutamate excitatory toxicity, conversely, BCAA supplements play a neuroprotective role in some diseases. Wang et al. [53] found that BCAA supplements enhanced the survival and axon regeneration of retinal ganglion cells in the optic nerve transverse injury model by activating the mTOR pathway. Further study indicated that BCAA supplements mitigated photoreceptor cell death in retinitis pigmentosa, inhibited RGC death, and promoted axon regeneration through promoting ATP production in glaucoma [54].

BCAAs also have a beneficial effect on traumatic brain injury (TBI) in animals and humans. Cole et al. [55] demonstrated that providing BCAAs in drinking water after TBI improved cognitive function as compared to untreated TBI mice. BCAA levels were significantly lower in TBI patients and were correlated with the severity of TBI [56]. BCAAs may affect cognitive recovery by increasing insulin levels and brain energy production. Increased brain energy availability can restore ionic homeostasis, reduce membrane depolarization and neuronal injury in ischemic regions [57,58]. In conclusion, the potential neuroprotective effects of BCAAs may come from a variety of reasons, including buffering toxic levels of glutamate, increasing glucose uptake and energy production, as well as activating mTOR.

### 4.3. Oxidative Stress

When organisms are exposed to biological and abiotic stressors such as hypoxia, an imbalance between oxidation and antioxidant occurs [59]. Oxidative stress is mainly characterized by elevated reactive oxygen species (ROS) levels. Excessive ROS results in morphological damage and functional weakening of DNA, protein, as well as lipids [60]. The retinal metabolism is active enough to maintain normal physiological functions; therefore, the retina produces more ROS by consuming a lot of oxygen. Under physiological conditions, appropriate ROS is beneficial to signal transduction in the retina [61]. However, overproduction of ROS is involved in the occurrence of DR [62] and AMD [63] by causing disturbances in cellular structure and function.

Ola et al. [20] used a new method for ROS measurement by injecting a fluorogenic marker into the eye cavities. ROS levels in the retina were measured 6 h after injection. Compared to the control rats, the ROS level was increased to almost 55% in the retinas of STZ-induced diabetic rats, and BCAA levels were also increased. BCATc inhibitor Gabapentin reduced the increasing level of BCAAs and decreased ROS levels to near control levels in the retinas of diabetic rats [20]. Excessive BCAA metabolism may lead to increased ROS production, resulting in RPE degeneration and AMD development. It has been reported that levels of isoleucine and leucine were dramatically enhanced in the retinas of leptin-deficient ob/ob mice. The ROS number was significantly increased by 70%, and levels of crucial oxidative stress genes were altered, including increasing H_2_O_2_ and glutathione peroxidase-3, as well as decreasing heme oxygenase-1. These results suggested that elevated oxidative stress in the ob/ob retina is associated with changes in metabolites such as BCAAs [44].

Moreover, BCATc inhibitor improved mitochondrial ROS production and mitochondrial membrane disruption, then participated in the occurrence and deterioration of non-alcoholic fatty liver disease [64]. BCAA metabolism can alleviate high-fat diet-induced oxidative stress and inflammation in the process of metabolic disorder [65]. BCAA treatment significantly increased levels of myocardial injury markers such as c-reactive protein and myocardial troponin in the heart. BCAAs also promoted the production of ROS and decreased the activity of superoxide dismutase. These results indicated that BCAA treatment resulted in myocardial injury through upregulation of ROS production [66]. Zhenyukh et al. [67] found that BCAAs increased the expression of genes associated with antioxidant defense, such as SOD1, SOD 2, CAT, and GPx1. An in vitro study reported that BCAAs promoted oxidative stress and the proinflammatory state of peripheral blood mononuclear cells in healthy donors through NADPH oxidase and mitochondrial communication [67].

### 4.4. Inflammation

Inflammation is an integrative series of physiological responses to foreign organisms such as dust particles, human pathogens, and viruses. There are many molecules involved in systemic inflammation that act as inflammatory markers, such as c-reactive protein and soluble intercellular adhesion molecule-1 [68]. Studies have shown that inflammation is a contributing regulatory factor involved in the development of both DR and AMD. Müller cells and astrocytes in the retina are involved in amplifying inflammatory responses by producing proinflammatory cytokines [69]. Levels of inflammatory factors, including IL-1β, IL-6, IL-8, TNF-α, and monocyte chemoattractant protein-1, were elevated in the ocular tissues of NPDR patients, especially IL-8 and TNF-α, compared with active PDR patients [70]. Complement components C3a and C5a are part of the drusen of AMD patients. Nozaki et al. [71] found that C3a and C5a were located in the drusen, RPE cells, as well as the Bruch membrane in AMD patients, which suggested complement activation in the development of AMD.

Gong et al. [19] found that BCAA-mediated mTOR signaling leads to inflammation during DR development. Levels of BCAAs and BCKA were increased in the retinas of db/db mice, with hyperglycemic time ranging from 4 to 12 weeks. Expressions of TNF-α, IL-6, intercellular adhesion molecule-1, and VEGF were promoted, and the mTOR pathway was activated when BCAAs accumulated. In the outer retinas of the ob/ob mice, there are distribution changes of C3-expression microglia and macrophages and enhancive complement activators C2 and Cfb, as well as a decreasing complement regulator Cfh. This study linked inflammation, complement activation, and BCAA metabolites in the retina of ob/ob mice. Understanding the relationship among these components in the retina contributes to the risk factor analysis of retinal degenerations [44].

According to a large cross-sectional study, higher circulating BCAA levels are related to the inflammatory markers above [68], and results from the individual BCAAs isoleucine, leucine, and valine showed a similar tendency to total BCAAs. Moreover, several studies explored the relationship between inflammation and circulating BCAA metabolites in humans [72,73]. A study containing 286 Finnish twins reported that the high sensitivity c-reactive protein levels were associated with leucine and isoleucine, but not valine [72]. However, there was no significant association between a high sensitivity c-reactive protein and serum BCAAs after the modulation of influencing factors including age, sex, smoking, and alcohol consumption in 611 Chinese adults [73]. This result could be due to the diverse ages of the participants and the lack of exclusion of prevalent T2D at blood draw.

BCAAs are also connected with neuroinflammation, referring to the natural immune response of the central nervous system. Associated molecular features of neuroinflammation include epigenetic mechanisms, synaptic plasticity deficits, decreased hippocampal neurogenesis, as well as altered expression of memory-related genes [74,75]. Persistent neuroinflammation is widely associated with neurodegenerative diseases. Existing studies have found that high doses of BCAAs changed the inflammatory profile in mixed glial cell cultures, the cerebral cortex, and hippocampus of rats [76,77]. BCAA supplements reduced the increasing IL-1β immunoreactivity caused by a high-fat diet in the hippocampus and affected astrocyte morphology, suggesting that BCAAs are involved in the regulation of obesity-induced neuroinflammatory responses [78].

### 4.5. Other Functions

The above functions are common in the retina and peripheral tissues. There are still some regulatory functions that are common in tissues but rarely reported in the retina. This may be related to the specific distribution of BCAA metabolic enzymes and key factors regulating function in tissues. Leucine participates in promoting muscle protein synthesis through activating mTOR and then regulating the phosphorylation of p70S6K and 4E-BP1 [79]. In the skeletal muscles, the optimal BCAAs ratio in a restricted protein diet promotes the level of uncoupling protein-3. Associated with glucose metabolism, uncoupling protein-3 abundance can regulate glucose uptake by regulating glucose transporter 4 (GLUT4) translocation [80]. The PI3K-AKT pathway that is also activated by BCAAs is then involved in regulating glucose metabolism [81].

Insulin is necessary to maintain glucose homeostasis. Dietary leucine supplements in drinking water were found to improve glucose tolerance and insulin sensitivity by decreasing tyrosine phosphorylation of insulin receptor substrate-1 (IRS-1) and decreasing insulin-stimulated serine phosphorylation of AKT then reducing hepatic steatosis [82]. On the other hand, giving BCAA mixtures to rats with a high-fat diet increased insulin resistance, which resulted from the activation of the mTOR/p70S6K pathway and phosphorylation of IRS1 on multiple serines [83]. White et al. [84] also showed that BCAA diet restriction recovers muscle insulin sensitivity in Zucker obese rats.

## 5. Mechanisms Responsible for Regulation of BCAA Catabolism and BCAA Function

BCAAs are widely used as dietary supplements to perform various functions. However, the mechanisms by which BCAAs play a role in diverse diseases are different. Therefore, this section sorted out and summarized the regulatory factors of BCAA metabolism and the downstream pathways regulated by BCAAs. BCAA accumulation is not only due to the changes in metabolic enzymes but may also be caused by the differences in other molecules, such as BCKDK, PPM1K, AMP-activated protein kinase (AMPK), and sodium-dependent glucose transporter 2 (SGLT2) inhibitor. BCAAs also perform a variety of biological functions by regulating downstream pathways including PI3K-AKT and mTOR.

### 5.1. Mechanisms Responsible for Regulation of BCAA Catabolism

#### 5.1.1. BCKDK

BCKDK inhibits BCKDH activity by the phosphorylation of three residues of BCKDHA. Conversely, BDKDK is negatively regulated by α-ketoacid, resulting in increased activation of BCKDH [85]. Tso et al. [86] found that KIC inhibits the activity of BCKDK by binding to an allosteric binding site of BCKDK. Increased BCKDH activity results in a reduction of BCAA concentration in the plasma with the presence of KIC in vivo [86]. BCKDK exerts a vital role in many serious diseases such as DR [19], maple syrup urine disease (MSUD), and obesity [87]. Using the analysis of whole exome-sequencing, the p.His162Gln variant of BCKDK was found in neonatal screening, showing a biochemical phenotype of mild MSUD. The mutation contributed to BCKDK activation by causing the conformational difference of BCKDK protein and decreasing the effect of inhibitors on BCKDK. Activation of BCKDK caused by the mutation resulted in inactivation of BCKDC and enhancive plasmatic BCAA levels [88].

#### 5.1.2. PPM1K

PPM1K is located at position 4q22.1 on chromosome 4 and results in a transcript of 1941 bp. Protein phosphatase 2Cm (PP2Cm) encoded by PPM1K can mediate the BCAA catabolism defect in diabetes [65]. PPM1K activates BCKDH by dephosphorylation of the E1α subunit and regulates BCAA metabolism. The inactivity of the BCKDH complex is closely related to the occurrence of the disease and is the main cause of autosomal recessive genetic disease MSUD. SNP array-based genotyping showed a loss of heterozygosity on chromosome 4 in patients with mild variant MSUD. PPM1K was selected for analysis and might be associated with the phenotype of patients. In the PPM1K (NM_152542.3) coding region, there was a homozygous c.417_418delTA change identified by Sanger sequencing. This mutation may result in the production of non-functional protein [89].

Higher BCAA levels in circulation are strongly responsible for a higher T2D risk [90,91]. According to genome-wide studies of BCAA levels, there were five genomic regions associated with BCAA level, of which significance with 21 kb upstream of the PPM1K was the strongest signal [90]. BCAAs are closely associated with SNPs rs1440581 and rs7678928 at the PPM1K locus; however, these two SNPs were in linkage disequilibrium with the rs1975393 in T2D [90]. In another study, Xuan et al. [91] found that the C-allele in rs1440581 was related to an increasing risk of T2D after adjustments for the confounder, according to logistic regression analyses. Rs1440581 significantly altered the influences of weight gain on increased fasting serum insulin and HOMA-IR, a homeostasis model evaluation indicator of insulin resistance. These results demonstrated that variant rs1440581 was relevant to a high risk of incident T2D and might modify the action of weight gain on insulin resistance [91].

#### 5.1.3. AMPK

As a crucial metabolic sensor, AMPK regulates energy homeostasis with the detection of changes in energy state. AMPK also can activate numerous targets coordinating changes in the production of energy. Upregulation of AMPK prevented various eye diseases, including DR, AMD, cataracts, and optic neuritis [92]. It has been reported that AMPK stimulation delays the photoreceptor degeneration induced by diabetes, with the regulation of the autophagy-related proteins LC3 and p62 expression. This process improves mitochondrion-related gene expression, membrane potential, and morphological abnormalities [92].

Exercise can lead to increased BCAA metabolism in skeletal muscle by activating AMPK [93]. AMPK regulates BCAA metabolism by increasing PPM1K and decreasing BCKDK expression, which will activate BCKDH and increase the flow of BCAAs to the TCA cycle [94]. Treatment of diabetic mice with pAMPK activator AICAR led to increased BCKDH activity, as well as decreased levels of BCAAs and BCKDK. Studies have shown that AICAR-mediated AMPK activation induces changes in the BCAA catabolic enzyme expression, playing a crucial role in enhancing BCAA metabolism in diabetic mice [94]. The expression of metabolic gene PGC-1α was decreased in diabetic patients and involved in regulating mitochondrial biogenesis and cell metabolism [95]. AMPK regulates PGC-1α activity through phosphorylation, and AICAR plays a regulatory role in a PGC1α-dependent manner, including stimulating pAMPK activation and mitochondrial biogenesis [96]. Excessive expression of PGC-1α in the skeletal muscle of mice led to increased BCAA metabolism and decreased BCAA levels by regulating BCKDH and BCAT [97,98]. Based on the above studies, AMPK activation directly induced the expression of PGC-1α and promoted the levels of BCAA catabolic enzyme regulated by PGC-1α.

#### 5.1.4. SGLT2 Inhibitor and L-Type Amino Acid Transporter (LAT1)

Gong et al. [19] found that SGLT2 inhibitor EMPA regulated the metabolic process of BCAAs. EMPA decreased the abnormal accumulation of BCAAs and BCKA, and decreased the level of BCKDK, while promoting the increase in BCKDH in the db/db retina. EMPA also led to inhibition of mTOR, downregulation of inflammation and angiogenesis factors, for example, IL-6, TNF-α, VCAM-1, as well as VEGF. BCAAs have difficulty entering the retina because of the existence of the blood–retinal barrier, which is important in maintaining the homeostatic microenvironment in the retina. The sodium-independent LAT1, as a large neutral amino acid (LNAA) transporter, is responsible for the transportation of BCAAs across the blood–retinal barrier [99,100], suggesting that LAT1 may be closely related to BCAA intake in the retina. Gong et al. [19] also found that LAT1 is expressed in the nerve fiber layer, ganglion cell layer, inner and outer plexiform layer, and RPE layers of the retina. Furthermore, elevated LAT1 expression was inhibited by EMPA. In conclusion, EMPA improves DR performance by regulating the normalization of intake and catabolism of BCAAs [19].

### 5.2. Mechanisms of BCAA Function

#### 5.2.1. PI3K-Akt Pathway

PI3K activates phosphoinositide-dependent kinase and atypical protein kinase C, then participates in glucose uptake via GLUT [101] as well as glycogen synthesis [102]. PDK activates the downstream effectors GLUT4, mTOR, and phosphodiesterase 3B by activating Akt, which is closely associated with subsequent glucose uptake, protein synthesis, and lipid inhibition [103]. The PI3K/Akt pathway participates in the pathogenesis of retinopathy, especially in DR and AMD, and can protect retinal photoreceptor cells and RPE from oxidative stress and inflammation mediated by high glucose in the DR model [104,105].

BCAAs have been demonstrated to regulate the PI3K/Akt pathway and elicit a variety of biological functions. Porcine jejunal epithelial cells were added to different concentrations of leucine, isoleucine, or valine; the results indicated that leucine treatment promoted the phosphorylation level of Akt in 5 min, and its level was higher at 40 and 60 min. The PI3K/Akt pathway was activated by the leucine treatment then regulated the expressions of peptide transporters and amino acids in the intestine [106]. BCAA deprivation also caused significant Akt activation by phosphorylating sites Ser473 or Thr308. Transcription factor KLF15 expression was promoted by BCAA starvation and Akt activation. KLF15 is a major regulate factor of the amino acid, glycemic, and lipid metabolism. These results indicated the role of BCAAs in metabolic regulation by regulating the PI3K/Akt pathway [107].

#### 5.2.2. mTOR Pathway

Amino acids play vital roles in cell physiology and disease. There are two main amino acid-sensing pathways, mTOR and nonderepressible 2 kinase (GCN2), to deal with their adequacy [108]. Uncharged tRNA activated GCN2 in conditions with limited amino acids and BCAA starvation. The eukaryotic translation initiation factor 2 subunit α was then activated by GCN2 through phosphorylation and subsequently inhibited protein synthesis as well as liver lipogenesis [109].

As a conservative protein kinase, mTOR is the main regulator of cell metabolism and responds to nutrients levels. The two protein forms, mTORC1 and mTORC2, are structurally and functionally different complexes [110]. mTORC1 performs a variety of biological functions including regulating autophagy and promoting protein synthesis. As a regulator of the insulin-related PI3K-Akt pathway, mTORC2 can respond to the growth factor signaling pathway by phosphorylating Akt [111]. Multiple crucial biological processes were closely associated with the mTOR signaling pathway, including autophagy, ribosome biogenesis, mitogenic signals, oxygen levels, cell growth and proliferation, energy status, and growth factors [112]. mTOR disorders have been identified in many diseases, including AMD, DR, retinitis pigmentosa, traumatic optic neuropathy, and glaucoma. Avet-Rochex et al. [113] found that the development of the retina and optic nerve in Drosophila is mainly regulated by mTOR pathway activity via influencing neurogenesis. Moreover, RGC differentiation in human or rat models was also temporally correlated with mTOR pathway activity [114]. mTOR also functions in vascular development through VEGF expression mediated by HIF-1α; this process can be delayed by inhibiting the mTORC1 disruption [115].

Recent studies have reported that BCAAs, especially leucine, exert a primary role in mTORC1 activation. Sestrin2 was identified as a leucine sensor for the mTORC1 signal pathway in cytoplasm [116]. Sestrin2 can bind to leucine and then be released from a complex with a GTPase-activating protein 2 (GATOR2), which activates mTORC1 as a positive regulator. GATOR2 interacts with GATOR1 on the lysosomal membrane, which activates a complex of Rag guanosine triphosphatases (GTPases) [117]. The process of GTP-RagA/B binding to mTOR regulatory-related proteins contributes to the mTORC1 translocation to the lysosome. The activated GTP-binding protein promotes the mTORC1 kinase activity in the lysosome [118]. mTORC1 activity is required for BCAA-induced protein anabolic signaling in the rodent, since the inhibitor of mTOR prevents leucine-induced increases in the phosphorylation of p70S6K and 4E-BP1 [119].

#### 5.2.3. Other Pathways

Excepting the PI3K-AKT and mTOR pathways mentioned above, it has been shown that BCAAs activate the NF-kB pathway then promote release of TNF-α and IL-6 in peripheral blood mononuclear cells [67]. The cGAS-STING pathway may also play a role in this process by promoting the NF-kB response [120]. Dietary L-isoleucine supplementation decreased the proportion of immune cells and reduced expressions of proinflammatory cytokines and levels of TLR4, MyD88, NF-kB in the colon. Results indicated that dietary supplementation with L-isoleucine participated in inhibiting inflammation in rats through regulating the TLR4/MyD88/NF-kB pathway, including amelioration of growth retardation induced by DSS and decreased colon injury [121]. BCAA treatment increased ileal β-defensins expression in weaned piglets, possibly through activating the Sirt1/ERK/90RSK signaling pathway. This result showed that BCAAs are also closely responsible for intestinal functions [122]. SLC25A44, a member of the mitochondrial membrane transporter family, was identified as the BCAA transporter in mitochondrial membranes [123]. BCAAs in the mitochondrion were used for thermogenesis in brown adipose tissue under cold environments. The active BCAA catabolism in brown adipose tissue was mediated by SLC25A44 and significantly affected thermogenic activity, energy expenditure, and overall metabolic health [124].

## 6. Conclusions

In recent years, BCAAs have been shown to be highly related to retinopathy, especially DR and AMD. We summarized the alterations in the metabolic differences of BCAAs in retinopathy to gain insight into the role of BCAAs in the retina. In addition, we included the functions and mechanisms of BCAAs in other metabolic tissues for reference, including muscle, liver, and adipose tissues. The BCAA metabolism is mainly controlled by upstream regulators such as BCKDK and PPM1K. BCAAs mainly stimulate the mTOR and PI3K-AKT signaling pathways to perform oxidative stress, inflammation, neuroprotection, and glucolipid metabolism in multiple metabolic tissues, which provides new ideas for exploring the roles and mechanisms of BCAAs in the retina. However, the currently available data are still limited to explaining how BCAA levels in other tissues or circulation contribute to the function of BCAAs in the retina. Therefore, studying the BCAA metabolism in different tissues will provide a better understanding of the future interaction of the tissue–retina axis.

## Data Availability

Not applicable.

## References

[1] GBD 2019 Blindness and Vision Impairment Collaborators, Vision Loss Expert Group of the Global Burden of Disease Study (2021). Trends in prevalence of blindness and distance and near vision impairment over 30 years: An analysis for the Global Burden of Disease Study. Lancet Glob. Health.

[2] Nathan D.M., Bebu I., Hainsworth D., Klein R., Tamborlane W., Lorenzi G., Gubitosi-Klug R., Lachin J.M., DCCT/EDIC Research Group (2017). Frequency of Evidence-Based Screening for Retinopathy in Type 1 Diabetes. N. Engl. J. Med..

[3] Fu Z., Usui-Ouchi A., Allen W., Tomita Y. (2022). Retinal Disease and Metabolism. Life.

[4] Simó-Servat O., Hernández C., Simó R. (2019). Diabetic Retinopathy in the Context of Patients with Diabetes. Ophthalmic. Res..

[5] Wong T.Y., Cheung C.M., Larsen M., Sharma S., Simó R. (2016). Diabetic retinopathy. Nat. Rev. Dis. Primers.

[6] Jian Q., Wu Y., Zhang F. (2022). Metabolomics in Diabetic Retinopathy: From Potential Biomarkers to Molecular Basis of Oxidative Stress. Cells.

[7] Grauslund J. (2017). Vascular endothelial growth factor inhibition for proliferative diabetic retinopathy: Et tu, Brute?. Acta Ophthalmol..

[8] Wong W.L., Su X., Li X., Cheung C.M.G., Klein R., Cheng C.Y., Wong T.Y. (2014). Global prevalence of age-related macular degeneration and disease burden projection for 2020 and 2040: A systematic review and meta-analysis. Lancet Glob. Health.

[9] Casten R.J., Rovner B.W. (2013). Update on depression and age-related macular degeneration. Curr. Opin. Ophthalmol..

[10] Thomas C.J., Mirza R.G., Gill M.K. (2021). Age-Related Macular Degeneration. Med. Clin. N. Am..

[11] Ferris F.L., Fine S.L., Hyman L. (1984). Age-related macular degeneration and blindness due to neovascular maculopathy. Arch. Ophthalmol..

[12] Chen Y., Coorey N.J., Zhang M., Zeng S., Madigan M.C., Zhang X., Gillies M.C., Zhu L., Zhang T. (2022). Metabolism Dysregulation in Retinal Diseases and Related Therapies. Antioxidants.

[13] Xia M., Zhang F. (2022). Amino Acids Metabolism in Retinopathy: From Clinical and Basic Research Perspective. Metabolites.

[14] Davis T.A., Fiorotto M.L., Reeds P.J. (1993). Amino acid compositions of body and milk protein change during the suckling period in rats. J. Nutr..

[15] Neinast M.D., Jang C., Hui S., Murashige D.S., Chu Q., Morscher R.J., Li X., Zhan L., White E., Anthony T.G. (2019). Quantitative Analysis of the Whole-Body Metabolic Fate of Branched-Chain Amino Acids. Cell Metab..

[16] Holeček M. (2018). Branched-chain amino acids in health and disease: Metabolism, alterations in blood plasma, and as supplements. Nutr. Metab..

[17] Lynch C.J., Adams S.H. (2014). Branched-chain amino acids in metabolic signalling and insulin resistance. Nat. Rev. Endocrinol..

[18] Wang R., Jian Q., Hu G., Du R., Xu X., Zhang F. (2022). Integrated Metabolomics and Transcriptomics Reveal Metabolic Patterns in Retina of STZ-Induced Diabetic Retinopathy Mouse Model. Metabolites.

[19] Gong Q., Zhang R., Wei F., Fang J., Zhang J., Sun J., Sun Q., Wang H. (2022). SGLT2 inhibitor-empagliflozin treatment ameliorates diabetic retinopathy manifestations and exerts protective effects associated with augmenting branched chain amino acids catabolism and transportation in db/db mice. Biomed. Pharmacother..

[20] Ola M.S., Alhomida A.S., LaNoue K.F. (2019). Gabapentin Attenuates Oxidative Stress and Apoptosis in the Diabetic Rat Retina. Neurotox. Res..

[21] Ichihara A., Koyama E. (1966). Transaminase of branched chain amino acids. I. Branched chain amino acids-alpha-ketoglutarate transaminase. J. Biochem..

[22] Ichihara A. (1975). Isozyme patterns of branched-chain amino acid transaminase during cellular differentiation and carcinogenesis. Ann. N. Y. Acad. Sci..

[23] Goto M., Shinno H., Ichihara A. (1977). Isozyme patterns of branched-chain amino acid transaminase in human tissues and tumors. Gan.

[24] Johnson W.A., Connelly J.L. (1972). Cellular localization and characterization of bovine liver branched-chain -keto acid dehydrogenases. Biochemistry.

[25] Neinast M., Murashige D., Arany Z. (2019). Branched Chain Amino Acids. Annu. Rev. Physiol..

[26] Rochfort S. (2005). Metabolomics reviewed: A new “omics” platform technology for systems biology and implications for natural products research. J. Nat. Prod..

[27] Jüppner J., Mubeen U., Leisse A., Caldana C., Brust H., Steup M., Herrmann M., Steinhauser D., Giavalisco P. (2017). Dynamics of lipids and metabolites during the cell cycle of *Chlamydomonas reinhardtii*. Plant J..

[28] Li Q., Wei S., Wu D., Wen C., Zhou J. (2018). Urinary Metabolomics Study of Patients with Gout Using Gas Chromatography-Mass Spectrometry. Biomed. Res. Int..

[29] Frayser R., Buse M.G. (1978). Branched chain amino acid metabolism in the retina of diabetic rats. Diabetologia.

[30] Valle D., Walser M., Brusilow S.W., Kaiser-Kupfer M. (1980). Gyrate atrophy of the choroid and retina: Amino acid metabolism and correction of hyperornithinemia with an arginine-deficient diet. J. Clin. Investig..

[31] Masser D.R., Starkey H.D.V., Bixler G.V., Dunton W., Bronson S.K., Freeman W.M. (2014). Insulin treatment normalizes retinal neuroinflammation but not markers of synapse loss in diabetic rats. Exp. Eye Res..

[32] Socha O., Dračínský M. (2020). Dimerization of Acetic Acid in the Gas Phase-NMR Experiments and Quantum-Chemical Calculations. Molecules.

[33] Chetwynd A.J., Dunn W.B., Rodriguez-Blanco G. (2017). Collection and Preparation of Clinical Samples for Metabolomics. Adv. Exp. Med. Biol..

[34] Yu Z., Kastenmüller G., He Y., Belcredi P., Möller G., Prehn C., Mendes J., Wahl S., Roemisch-Margl W., Ceglarek U. (2011). Differences between human plasma and serum metabolite profiles. PLoS ONE.

[35] Holekamp N.M. (2010). The vitreous gel: More than meets the eye. Am. J. Ophthalmol..

[36] Yalcinbayir O., Buyukuysal R.L., Gelisken O., Buyukuysal C., Can B. (2014). Amino acid and vascular endothelial growth factor levels in subretinal fluid in rhegmatogenous retinal detachment. Mol. Vis..

[37] Bertram K.M., Bula D.V., Pulido J.S., Shippy S.A., Gautam S., Lu M.J., Hatfield R.M., Kim J.H., Quirk M.T., Arroyo J.G. (2008). Amino-acid levels in subretinal and vitreous fluid of patients with retinal detachment. Eye.

[38] Wang H., Fang J., Chen F., Sun Q., Xu X., Lin S.H., Liu K. (2020). Metabolomic profile of diabetic retinopathy: A GC-TOFMS-based approach using vitreous and aqueous humor. Acta Diabetol..

[39] Xuan Q., Ouyang Y., Wang Y., Wu L., Li H., Luo Y., Zhao X., Feng D., Qin W., Hu C. (2020). Multiplatform Metabolomics Reveals Novel Serum Metabolite Biomarkers in Diabetic Retinopathy Subjects. Adv. Sci..

[40] Mendez K.M., Kim J., Laíns I., Nigalye A., Katz R., Pundik S., Kim I.K., Liang L., Vavvas D.G., Miller J.B. (2021). Association of Human Plasma Metabolomics with Delayed Dark Adaptation in Age-Related Macular Degeneration. Metabolites.

[41] Lin H.-T., Cheng M.-L., Lo C.-J., Lin G., Lin S.-F., Yeh J.-T., Ho H.-Y., Lin J.-R., Liu F.-C. (2019). 1H Nuclear Magnetic Resonance (NMR)-Based Cerebrospinal Fluid and Plasma Metabolomic Analysis in Type 2 Diabetic Patients and Risk Prediction for Diabetic Microangiopathy. J. Clin. Med..

[42] Gowda K., Zinnanti W.J., LaNoue K.F. (2011). The influence of diabetes on glutamate metabolism in retinas. J. Neurochem..

[43] Zhou Y., Tan W., Zou J., Cao J., Huang Q., Jiang B., Yoshida S., Li Y. (2021). Metabolomics Analyses of Mouse Retinas in Oxygen-Induced Retinopathy. Investig. Ophthalmol. Vis. Sci..

[44] Natoli R., Fernando N., Dahlenburg T., Jiao H., Aggio-Bruce R., Barnett N.L., De La Barca J.M.C., Tcherkez G., Reynier P., Fang J. (2018). Obesity-induced metabolic disturbance drives oxidative stress and complement activation in the retinal environment. Mol. Vis..

[45] Wei P., He M., Han G. (2022). Metabolic Characterization of Ocular Tissues in Relation to Laser-Induced Choroidal Neovascularization in Rats. J. Proteome Res..

[46] Xu R., Ritz B.K., Wang Y., Huang J., Zhao C., Gong K., Liu X., Du J. (2020). The retina and retinal pigment epithelium differ in nitrogen metabolism and are metabolically connected. J. Biol. Chem..

[47] Gibson C.J. (1988). Diurnal alterations in retinal tyrosine level and dopamine turnover in diabetic rats. Brain. Res..

[48] Elmi A., Ventrella D., Laghi L., Carnevali G., Zhu C., Pertile G., Barone F., Benfenati F., Bacci M.L. (2019). ^1^H NMR Spectroscopy Characterization of Porcine Vitreous Humor in Physiological and Photoreceptor Degeneration Conditions. Investig. Ophthalmol. Vis. Sci..

[49] Herman M.A., She P., Peroni O.D., Lynch C.J., Kahn B.B. (2010). Adipose tissue branched chain amino acid (BCAA) metabolism modulates circulating BCAA levels. J. Biol. Chem..

[50] Zhou Y., Danbolt N.C. (2014). Glutamate as a neurotransmitter in the healthy brain. J. Neural Transm..

[51] Lieth E., LaNoue K.F., Berkich D.A., Xu B., Ratz M., Taylor C., Hutson S.M. (2001). Nitrogen shuttling between neurons and glial cells during glutamate synthesis. J. Neurochem..

[52] Contrusciere V., Paradisi S., Matteucci A., Malchiodi-Albedi F. (2010). Branched-chain amino acids induce neurotoxicity in rat cortical cultures. Neurotox. Res..

[53] Wang T., Wei X.Y., Yang A.A., Liu Z., Wang S.Q., You Y.Y., Kuang F., You S.W., Wu M.M. (2018). Branched-Chain Amino Acids Enhance Retinal Ganglion Cell Survival and Axon Regeneration after Optic Nerve Transection in Rats. Curr. Eye Res..

[54] Hasegawa T., Ikeda H.O., Iwai S., Muraoka Y., Tsuruyama T., Okamoto-Furuta K., Kohda H., Kakizuka A., Yoshimura N. (2018). Branched chain amino acids attenuate major pathologies in mouse models of retinal degeneration and glaucoma. Heliyon.

[55] Cole J.T., Mitala C.M., Kundu S., Verma A., Elkind J.A., Nissim I., Cohen A.S. (2010). Dietary branched chain amino acids ameliorate injury-induced cognitive impairment. Proc. Natl. Acad. Sci. USA.

[56] Jeter C.B., Hergenroeder G.W., Ward N.H., Moore A.N., Dash P.K. (2013). Human mild traumatic brain injury decreases circulating branched-chain amino acids and their metabolite levels. J. Neurotrauma.

[57] Mattson M.P. (1997). Neuroprotective signal transduction, relevance to stroke. Neurosci. Biobehav. Rev..

[58] Paschen W., Frandsen A. (2001). Endoplasmic reticulum dysfunction--a common denominator for cell injury in acute and degenerative diseases of the brain?. J. Neurochem..

[59] Zhu L., Zang J., Liu B., Yu G., Hao L., Liu L., Zhong J. (2020). Oxidative stress-induced RAC autophagy can improve the HUVEC functions by releasing exosomes. J. Cell. Physiol..

[60] Guo C., Sun L., Chen X., Zhang D. (2013). Oxidative stress, mitochondrial damage and neurodegenerative diseases. Neural Regen. Res..

[61] Datta S., Cano M., Ebrahimi K., Wang L., Handa J.T. (2017). The impact of oxidative stress and inflammation on RPE degeneration in non-neovascular AMD. Prog. Retin. Eye Res..

[62] Kang Q., Yang C. (2020). Oxidative stress and diabetic retinopathy: Molecular mechanisms, pathogenetic role and therapeutic implications. Redox. Biol..

[63] Kaarniranta K., Pawlowska E., Szczepanska J., Jablkowska A., Blasiak J. (2019). Role of Mitochondrial DNA Damage in ROS-Mediated Pathogenesis of Age-Related Macular Degeneration (AMD). Int. J. Mol. Sci..

[64] Lu Z., Sun G.F., Pan X.A., Qu X.H., Yang P., Chen Z.P., Han X.J., Wang T. (2022). BCATc inhibitor 2 ameliorated mitochondrial dysfunction and apoptosis in oleic acid-induced non-alcoholic fatty liver disease model. Front. Pharmacol..

[65] Lian K., Guo X., Wang Q., Liu Y., Wang R.-T., Gao C., Li G.-Y., Li C.-X., Tao L. (2020). PP2Cm overexpression alleviates MI/R injury mediated by a BCAA catabolism defect and oxidative stress in diabetic mice. Eur. J. Pharmacol..

[66] Jiang Y.-J., Sun S.-J., Cao W.-X., Lan X.-T., Ni M., Fu H., Li D.-J., Wang P., Shen F.-M. (2021). Excessive ROS production and enhanced autophagy contribute to myocardial injury induced by branched-chain amino acids: Roles for the AMPK-ULK1 signaling pathway and α7nAChR. Biochim. Biophys. Acta Mol. Basis. Dis..

[67] Zhenyukh O., Civantos E., Ruiz-Ortega M., Sánchez M.S., Vázquez C., Peiró C., Egido J., Mas S. (2017). High concentration of branched-chain amino acids promotes oxidative stress, inflammation and migration of human peripheral blood mononuclear cells via mTORC1 activation. Free Radic. Biol. Med..

[68] Hamaya R., Mora S., Lawler P.R., Cook N.R., Ridker P.M., Buring J.E., Lee I.-M., Manson J.E., Tobias D.K. (2021). Association of Plasma Branched-Chain Amino Acid With Biomarkers of Inflammation and Lipid Metabolism in Women. Circ. Genom. Precis. Med..

[69] Sorrentino F.S., Allkabes M., Salsini G., Bonifazzi C., Perri P. (2016). The importance of glial cells in the homeostasis of the retinal microenvironment and their pivotal role in the course of diabetic retinopathy. Life Sci..

[70] Boss J.D., Singh P.K., Pandya H.K., Tosi J., Kim C., Tewari A., Juzych M.S., Abrams G.W., Kumar A. (2017). Assessment of Neurotrophins and Inflammatory Mediators in Vitreous of Patients With Diabetic Retinopathy. Investig. Ophthalmol. Vis. Sci..

[71] Nozaki M., Raisler B.J., Sakurai E., Sarma J.V., Barnum S.R., Lambris J.D., Chen Y., Zhang K., Ambati B.K., Baffi J.Z. (2006). Drusen complement components C3a and C5a promote choroidal neovascularization. Proc. Natl. Acad. Sci. USA.

[72] Bogl L.H., Kaye S.M., Rämö J.T., Kangas A.J., Soininen P., Hakkarainen A., Lundbom J., Lundbom N., Ortega-Alonso A., Rissanen A. (2016). Abdominal obesity and circulating metabolites: A twin study approach. Metabolism.

[73] Sun L., Hu C., Yang R., Lv Y., Yuan H., Liang Q., He B., Pang G., Jiang M., Dong J. (2017). Association of circulating branched-chain amino acids with cardiometabolic traits differs between adults and the oldest-old. Oncotarget.

[74] Fischer A., Sananbenesi F., Wang X., Dobbin M., Tsai L.-H. (2007). Recovery of learning and memory is associated with chromatin remodelling. Nature.

[75] Heyward F.D., Gilliam D., Coleman M.A., Gavin C.F., Wang J., Kaas G., Trieu R., Lewis J., Moulden J., Sweatt J.D. (2016). Obesity Weighs down Memory through a Mechanism Involving the Neuroepigenetic Dysregulation of Sirt1. J. Neurosci..

[76] De Simone R., Vissicchio F., Mingarelli C., De Nuccio C., Visentin S., Ajmone-Cat M.A., Minghetti L. (2013). Branched-chain amino acids influence the immune properties of microglial cells and their responsiveness to pro-inflammatory signals. Biochim. Biophys. Acta.

[77] Rosa L., Scaini G., Furlanetto C.B., Galant L.S., Vuolo F., Dall’Igna D.M., Schuck P.F., Ferreira G.C., Dal-Pizzol F., Streck E.L. (2016). Administration of branched-chain amino acids alters the balance between pro-inflammatory and anti-inflammatory cytokines. Int. J. Dev. Neurosci..

[78] Feijo GD S., Jantsch J., Correia L.L., Eller S., Furtado-Filho O.V., Giovenardi M., Porawski M., Braganhol E., Guedes R.P. (2022). Neuroinflammatory responses following zinc or branched-chain amino acids supplementation in obese rats. Metab. Brain Dis..

[79] Murgas Torrazza R., Suryawan A., Gazzaneo M.C., Orellana R.A., Frank J.W., Nguyen H.V., Fiorotto M.L., El-Kadi S., Davis T.A. (2010). Leucine supplementation of a low-protein meal increases skeletal muscle and visceral tissue protein synthesis in neonatal pigs by stimulating mTOR-dependent translation initiation. J. Nutr..

[80] Duan Y., Li F., Wang W., Guo Q., Wen C., Yin Y. (2017). Alteration of muscle fiber characteristics and the AMPK-SIRT1-PGC-1α axis in skeletal muscle of growing pigs fed low-protein diets with varying branched-chain amino acid ratios. Oncotarget.

[81] Nie C., He T., Zhang W., Zhang G., Ma X. (2018). Branched Chain Amino Acids: Beyond Nutrition Metabolism. Int. J. Mol. Sci..

[82] Macotela Y., Emanuelli B., Bång A.M., Espinoza D.O., Boucher J., Beebe K., Gall W., Kahn C.R. (2011). Dietary leucine—An environmental modifier of insulin resistance acting on multiple levels of metabolism. PLoS ONE.

[83] Newgard C.B., An J., Bain J.R., Muehlbauer M.J., Stevens R.D., Lien L.F., Haqq A.M., Shah S.H., Arlotto M., Slentz C.A. (2009). A branched-chain amino acid-related metabolic signature that differentiates obese and lean humans and contributes to insulin resistance. Cell Metab..

[84] White P.J., Lapworth A.L., An J., Wang L., McGarrah R.W., Stevens R.D., Ilkayeva O., George T., Muehlbauer M.J., Bain J.R. (2016). Branched-chain amino acid restriction in Zucker-fatty rats improves muscle insulin sensitivity by enhancing efficiency of fatty acid oxidation and acyl-glycine export. Mol. Metab..

[85] Chuang J.L., Wynn R.M., Chuang D.T. (2002). The C-terminal hinge region of lipoic acid-bearing domain of E2b is essential for domain interaction with branched-chain alpha-keto acid dehydrogenase kinase. J. Biol. Chem..

[86] Tso S.-C., Qi X., Gui W.-J., Chuang J.L., Morlock L.K., Wallace A.L., Ahmed K., Laxman S., Campeau P.M., Lee B.H. (2013). Structure-based design and mechanisms of allosteric inhibitors for mitochondrial branched-chain α-ketoacid dehydrogenase kinase. Proc. Natl. Acad. Sci. USA.

[87] Burrage L.C., Nagamani S.C., Campeau P.M., Lee B.H. (2014). Branched-chain amino acid metabolism: From rare Mendelian diseases to more common disorders. Hum. Mol. Genet..

[88] Maguolo A., Rodella G., Giorgetti A., Nicolodi M., Ribeiro R., Dianin A., Cantalupo G., Monge I., Carcereri S., De Bernardi M.L. (2022). A Gain-of-Function Mutation on BCKDK Gene and Its Possible Pathogenic Role in Branched-Chain Amino Acid Metabolism. Genes.

[89] Oyarzabal A., Martínez-Pardo M., Merinero B., Navarrete R., Desviat L.R., Ugarte M., Rodríguez-Pombo P. (2013). A novel regulatory defect in the branched-chain α-keto acid dehydrogenase complex due to a mutation in the PPM1K gene causes a mild variant phenotype of maple syrup urine disease. Hum. Mutat..

[90] Lotta L.A., Scott R.A., Sharp S.J., Burgess S., Luan J., Tillin T., Schmidt A.F., Imamura F., Stewart I.D., Perry J.R.B. (2016). Genetic Predisposition to an Impaired Metabolism of the Branched-Chain Amino Acids and Risk of Type 2 Diabetes: A Mendelian Randomisation Analysis. PLoS Med..

[91] Xuan L., Hou Y., Wang T., Li M., Zhao Z., Lu J., Xu Y., Chen Y., Qi L., Wang W. (2018). Association of branched chain amino acids related variant rs1440581 with risk of incident diabetes and longitudinal changes in insulin resistance in Chinese. Acta Diabetol..

[92] Song S., Bao S., Zhang C., Zhang J., Lv J., Li X., Chudhary M., Ren X., Kong L. (2021). Stimulation of AMPK Prevents Diabetes-Induced Photoreceptor Cell Degeneration. Oxid. Med. Cell Longev..

[93] Shimomura Y., Murakami T., Nakai N., Nagasaki M., Harris R.A. (2004). Exercise promotes BCAA catabolism: Effects of BCAA supplementation on skeletal muscle during exercise. J. Nutr..

[94] Lian K., Du C., Liu Y., Zhu D., Yan W., Zhang H., Hong Z., Liu P., Zhang L., Pei H. (2015). Impaired adiponectin signaling contributes to disturbed catabolism of branched-chain amino acids in diabetic mice. Diabetes.

[95] Wu Z., Puigserver P., Andersson U., Zhang C., Adelmant G., Mootha V., Troy A., Cinti S., Lowell B., Scarpulla R.C. (1999). Mechanisms controlling mitochondrial biogenesis and respiration through the thermogenic coactivator PGC-1. Cell.

[96] Jäger S., Handschin C., St-Pierre J., Spiegelman B.M. (2007). AMP-activated protein kinase (AMPK) action in skeletal muscle via direct phosphorylation of PGC-1alpha. Proc. Natl. Acad. Sci. USA.

[97] Sugimoto T., Uchitomi R., Hatazawa Y., Miura S., Kamei Y. (2021). Metabolomic analysis on blood of transgenic mice overexpressing PGC-1α in skeletal muscle. Biosci. Biotechnol. Biochem..

[98] Hatazawa Y., Tadaishi M., Nagaike Y., Morita A., Ogawa Y., Ezaki O., Takai-Igarashi T., Kitaura Y., Shimomura Y., Kamei Y. (2014). PGC-1α-mediated branched-chain amino acid metabolism in the skeletal muscle. PLoS ONE.

[99] Tomi M., Mori M., Tachikawa M., Katayama K., Terasaki T., Hosoya K.I. (2005). L-type amino acid transporter 1-mediated L-leucine transport at the inner blood-retinal barrier. Investig. Ophthalmol. Vis. Sci..

[100] Akanuma S.-I., Yamakoshi A., Sugouchi T., Kubo Y., Hartz A.M.S., Bauer B., Hosoya K.-I. (2018). Role of l-Type Amino Acid Transporter 1 at the Inner Blood-Retinal Barrier in the Blood-to-Retina Transport of Gabapentin. Mol. Pharm..

[101] Huang P., Frohman M.A. (2003). The role of phospholipase D in Glut-4 translocation. Diabetes Metab. Res. Rev..

[102] Shao C.Y., Crary J.F., Rao C., Sacktor T.C., Mirra S.S. (2006). A typical protein kinase C in neurodegenerative disease II: PKCiota/lambda in tauopathies and alpha-synucleinopathies. J. Neuropathol. Exp. Neurol..

[103] Thong F.S., Bilan P.J., Klip A. (2007). The Rab GTPase-activating protein AS160 integrates Akt, protein kinase C, and AMP-activated protein kinase signals regulating GLUT4 traffic. Diabetes.

[104] Lai T.T., Yang C.M., Yang C.H. (2020). Astaxanthin Protects Retinal Photoreceptor Cells against High Glucose-Induced Oxidative Stress by Induction of Antioxidant Enzymes via the PI3K/Akt/Nrf2 Pathway. Antioxidants.

[105] Janani R., Anitha R.E., Divya P., Chonche M., Baskaran V. (2022). Astaxanthin ameliorates hyperglycemia induced inflammation via PI3K/Akt-NF-κB signaling in ARPE-19 cells and diabetic rat retina. Eur. J. Pharmacol..

[106] Zhang S., Ren M., Zeng X., He P., Ma X., Qiao S. (2014). Leucine stimulates ASCT2 amino acid transporter expression in porcine jejunal epithelial cell line (IPEC-J2) through PI3K/Akt/mTOR and ERK signaling pathways. Amino Acids.

[107] Liu Y., Dong W., Shao J., Wang Y., Zhou M., Sun H. (2017). Branched-Chain Amino Acid Negatively Regulates KLF15 Expression via PI3K-AKT Pathway. Front. Physiol..

[108] Kimball S.R. (2001). Regulation of translation initiation by amino acids in eukaryotic cells. Prog. Mol. Subcell. Biol..

[109] Towle H.C. (2007). The metabolic sensor GCN2 branches out. Cell Metab..

[110] Bonvini A., Coqueiro A.Y., Tirapegui J., Calder P.C., Rogero M.M. (2018). Immunomodulatory role of branched-chain amino acids. Nutr. Rev..

[111] Saxton R.A., Sabatini D.M. (2017). mTOR Signaling in Growth, Metabolism, and Disease. Cell.

[112] Sarbassov D.D., Ali S.M., Sabatini D.M. (2005). Growing roles for the mTOR pathway. Curr. Opin. Cell Biol..

[113] Avet-Rochex A., Carvajal N., Christoforou C.P., Yeung K., Maierbrugger K.T., Hobbs C., Lalli G., Cagin U., Plachot C., McNeill H. (2014). Unkempt is negatively regulated by mTOR and uncouples neuronal differentiation from growth control. PLoS Genet..

[114] Teotia P., Van Hook M.J., Fischer D., Ahmad I. (2019). Human retinal ganglion cell axon regeneration by recapitulating developmental mechanisms: Effects of recruitment of the mTOR pathway. Development.

[115] Yagasaki R., Nakahara T., Mori A., Sakamoto K., Ishii K. (2014). Effects of mTOR inhibition on normal retinal vascular development in the mouse. Exp. Eye Res..

[116] Wolfson R.L., Chantranupong L., Saxton R.A., Shen K., Scaria S.M., Cantor J.R., Sabatini D.M. (2016). Sestrin2 is a leucine sensor for the mTORC1 pathway. Science.

[117] Martina J.A., Puertollano R. (2013). Rag GTPases mediate amino acid-dependent recruitment of TFEB and MITF to lysosomes. J. Cell Biol..

[118] Zoncu R., Bar-Peled L., Efeyan A., Wang S., Sancak Y., Sabatini D.M. (2011). mTORC1 senses lysosomal amino acids through an inside-out mechanism that requires the vacuolar H(+)-ATPase. Science.

[119] Anthony J.C., Yoshizawa F., Anthony T.G., Vary T.C., Jefferson L.S., Kimball S.R. (2000). Leucine stimulates translation initiation in skeletal muscle of postabsorptive rats via a rapamycin-sensitive pathway. J. Nutr..

[120] Balka K.R., Louis C., Saunders T.L., Smith A.M., Calleja D.J., D’Silva D.B., Moghaddas F., Tailler M., Lawlor K.E., Zhan Y. (2020). TBK1 and IKKε Act Redundantly to Mediate STING-Induced NF-κB Responses in Myeloid Cells. Cell Rep..

[121] Mao X., Sun R., Wang Q., Chen D., Yu B., He J., Yu J., Luo J., Luo Y., Yan H. (2021). l-Isoleucine Administration Alleviates DSS-Induced Colitis by Regulating TLR4/MyD88/NF-κB Pathway in Rats. Front. Immunol..

[122] Ren M., Zhang S., Liu X., Li S., Mao X., Zeng X., Qiao S. (2016). Different Lipopolysaccharide Branched-Chain Amino Acids Modulate Porcine Intestinal Endogenous β-Defensin Expression through the Sirt1/ERK/90RSK Pathway. J. Agric. Food Chem..

[123] Sun H., Wang Y. (2019). A new branch connecting thermogenesis and diabetes. Nat. Metab..

[124] Yoneshiro T., Wang Q., Tajima K., Matsushita M., Maki H., Igarashi K., Dai Z., White P.J., McGarrah R.W., Ilkayeva O.R. (2019). BCAA catabolism in brown fat controls energy homeostasis through SLC25A44. Nature.

